# Impact of Waiting Response Evaluation to First-Line Systemic Therapy before Considering Local Ablative Therapy in Metastatic Non-Small-Cell Lung Cancer

**DOI:** 10.3390/cancers15215127

**Published:** 2023-10-25

**Authors:** Lahcene Belaidi, Pascal Wang, Kevin Quintin, Catherine Durdux, Etienne Giroux-Leprieur, Philippe Giraud

**Affiliations:** 1 Department of Radiation Oncology, Hôpital Européen Georges Pompidou AP-HP, 20 Rue Leblanc, 75015 Paris, France; 2 Department of Pulmonology and Thoracic Oncology Service, Hôpital Ambroise Paré, 9 Av. Charles de Gaulle, 92100 Boulogne-Billancourt, France

**Keywords:** non-small-cell lung cancer, stereotactic ablative radiotherapy, oligometastatic disease, oligoprogressive disease, locally ablative therapy, sequence, immune checkpoint inhibitor

## Abstract

**Simple Summary:**

Stereotactic radiotherapy (SRT) is becoming increasingly important in managing metastatic non-small-cell lung cancer (mNSCLC). However, the optimal timing of SRT in relation to systemic treatment remains unclear. Herein, we aimed to determine whether waiting response evaluation to first-line systemic therapy (FLST) before considering local SRT treatment could exclude poorer prognosis progressive tumor that may not benefit from SRT. We compared survival data for 50 patients locally treated before or within 90 days of initiating FLST (early SRT), with 49 patients treated at least 90 days after initiating FLST (late SRT). In patients receiving conventional chemotherapy, late SRT resulted in significantly better survival outcomes compared to early SRT. For patients receiving an immune checkpoint inhibitor (ICI), there was no difference between late and early SRT. These results suggest that delaying SRT treatment may be less necessary when ICI is administered in mNSCLC

**Abstract:**

Stereotactic radiotherapy (SRT) is gaining increasing importance in metastatic non-small-cell lung cancer (mNSCLC) management. The optimal sequence of tumor irradiation relative to systemic treatment remains unclear. If waiting response evaluation to first-line systemic therapy (FLST) before considering local treatment may allow for the exclusion of poorer prognosis progressive tumors that may not benefit from SRT, performing irradiation near immune check point inhibitor (ICI) first administration seems to improve their synergic effect. Herein, we aimed to determine whether delaying SRT after response evaluation to FLST would result in better prognosis. We compared overall survival (OS), progression-free survival (PFS), and time to first subsequent therapy (TFST) for 50 patients locally treated before or within 90 days of initiating FLST (early SRT), with 49 patients treated at least 90 days after initiating FLST (late SRT). Patients treated with conventional chemotherapy alone exhibited significantly poorer median OS, PFS, and TFST in the early SRT arm: (in months) 16.5 [8.33-NR] vs. 58.3 [35.05-NR] (*p* = 0.0015); 4.69 [3.57–8.98] vs. 8.20 [6.66–12.00] (*p* = 0.017); and 6.26 [4.82–11.8] vs. 10.0 [7.44–21.8] (*p* = 0.0074), respectively. Patient receiving ICI showed no difference in OS (NR [25.2-NR] vs. 36.6 [35.1-NR], *p* = 0.79), PFS (7.54 [6.23-NR] vs. 4.07 [2.52-NR], *p* = 0.19), and TFST (13.7 [9.48-NR] vs. 10.3 [3.54-NR], *p* = 0.49). These results suggest that delaying SRT treatment in order to filter a rapidly growing tumor may be less necessary when ICI is administered in mNSCLC.

## 1. Introduction

Locally ablative treatment (LAT) is gaining increasing importance in metastatic cancer management. In low-burden diseases (usually defined as “oligometastatic diseases” by setting a maximum limit of 3 to 5 lesions and a maximum limit of 3 different organ systems affected [1,2,3,4]), it has been demonstrated that LAT with stereotactic ablative radiotherapy (SRT) on all metastatic lesions results in a significant improvement in overall survival (OS) and progression-free survival (PFS) compared to systemic therapy alone [5]. Long-term data even suggest the potential for long-term survival [6].

In the specific context of metastatic non-small-cell lung cancer (mNSCLC), several prospective randomized trials have also shown a significant benefit from local treatment [7,8,9,10,11,12]. However, the sequence of systemic treatment administration relative to SRT differs between study designs, and the optimal approach remains unclear [13,14]. Trials using only chemotherapy drugs as systemic treatment suggest reserving LAT for patients who do not progress after first-line systemic therapy (FLST) [7,8,9,10]. Therefore, local treatment is delayed in order to exclude poorer prognosis progressive tumors that may not benefit from SRT. On the other hand, trials using immune check point inhibitor (ICI) as systemic treatment have typically performed LAT before evaluating tumor response to systemic treatment [15,16]. This approach may be driven by the fact that at the time of the study design, the safety of combining LAT and ICI was not yet established. However, available preclinical and phase I data suggest that performing SRT slightly before or near the first administration of ICI may be the optimal sequence to induce an abscopal antitumor immune response [17,18,19,20,21,22,23,24,25,26,27,28,29]. The timing of local tumor irradiation might therefore have a significant impact on the efficacy of ICI treatment. 

In this study, our aim was to determine whether waiting for response evaluation to first-line systemic therapy before considering local ablative therapy in metastatic non-small-cell lung cancer could lead to differences in overall survival (OS), progression-free survival (PFS), and time to first subsequent therapy (TFST) for patients treated by SRT for mNSCLC.

## 2. Materials and Methods

### 2.1. Population

This retrospective, monocentric study included patients treated in an academic hospital radiotherapy department between 2016 and 2021 for an mNSCLC.

They were 18 years or older and received SRT treatment on at least one metastatic site and one line of systemic treatment. All tumors were epidermal growth factor receptor (EGFR), anaplastic lymphoma kinase (ALK), and c-ros oncogene 1 (ROS-1) wild-type. Systemic treatment could be either platinum doublet chemotherapy, anti-PD1/PDL1 monoclonal antibodies, or a combination of both. 

Follow-up by medical oncologist was ensured in six different institutions.

The study was approved by the institutional review board (Comité d’éthique de la recherche, Assistance Publique-Hôpitaux de Paris).

### 2.2. Treatment Sequences

The first group of patients, referred to as “early SRT”, was defined by an SRT 1st session performed before or within 90 days after the initiation of first-line systemic treatment. We assumed these patients underwent local treatment before the assessment of tumor response to first-line systemic treatment. 

The second group of patients, referred to as “late SRT”, was defined by an SRT 1st session performed at least 90 days after the initiation of first-line systemic treatment. We assumed these patients underwent local treatment after the assessment of tumor response to first-line systemic treatment. This could either be consolidative SRT, if no lesions were progressing, or oligoprogressive SRT if there were 1 to 5 oligoprogressive lesions. 

We opted for the 90-day delay as it aligns with the protocol outlined in the studies conducted by Gomez et al. [7,8] and Lyengar et al. [9]. Additionally, this 90-day period is typically when assessments of systemic therapy response are conducted in clinical practice.

SRT was performed using an M6 Cyberknife Robotic Radiosurgery System (Accuray Incorporated, Sunnyvale, CA, USA). Cumulative SRT dose for brain lesions was 27 Gy for 3 fraction schedules and 30 Gy for 5 fraction schedules. Cumulative SRT doses for lungs lesions were 60 Gy for 3, 5, and 8 fraction schedules. A total of 36 Gy in 6 fractions was accepted if dose constraints could not be met with a 60 Gy cumulative dose.

Choices of systemic treatment were determined by the medical oncology team.

### 2.3. Outcomes

The primary outcome was overall survival (OS) defined from the time of metastatic disease diagnosis to the time of death. Secondary outcomes were progression-free survival (PFS) and time to first subsequent therapy (TFST). 

PFS was defined from the time of first systemic treatment administration to the time of disease progression or death, whichever occurred first. 

TFST was defined from the time of first systemic treatment administration to time of initiation of second line systemic treatment or death, whichever occurred first. Data were obtained by reviewing computerized records.

Patients had at least one PET-CT, CT-scan, and brain imaging at disease diagnosis and then had a radiographic evaluation every 12 weeks (by CT +/− TEP and brain imaging).

### 2.4. Subgroup Analysis 

We aimed to investigate these outcomes in the overall population, as well as in two distinct subgroups: a subgroup of patients who received chemotherapy only as their first-line systemic treatment, and a subgroup of patients who received at least one ICI drug as their first-line systemic therapy, either as monotherapy or in combination with conventional chemotherapy.

Furthermore, we conducted additional exploratory analyses on the following subgroups: patients with synchronous diseases (defined as the first metastasis diagnosed within 3 months of the primary cancer), patients with metachronous diseases (defined as the first metastasis diagnosed more than 3 months after the diagnosis of the primary cancer), patients with more than 3 metastases at diagnosis, and patients with 3 or fewer metastases at diagnosis.

### 2.5. Statistical Analyses

Statistical analyses were conducted using R software version 4.2.2. 

The Kaplan–Meier method was used to estimate overall survival, progression-free survival, and time to first subsequent therapy. 

The stratified log-rank test was used to assess between-group differences. Hazard ratios and their associated 95% confidence intervals (CI) were calculated using a stratified Cox proportional-hazards model for univariate and multivariate analysis.

Interaction effects tests were conducted using a likelihood ratio test.

Follow-up times were calculated using the reverse Kaplan–Meier method. 

The statistical significance threshold was set at 0.05.

## 3. Results

### 3.1. Population Description

In total, 99 patients were included in the main analysis. A total of 50 patients (51%) were locally treated with SRT before or within 90 days after the initiation of first-line systemic therapy (early SRT), and 49 patients (49%) were treated with SRT at least 90 days after the initiation of first-line systemic therapy (late SRT). Median follow-up was 41.2 months [95% CI: 32.8–53.5]. 

A total of 30 patients (30%) received at least one ICI drug as first-line systemic therapy, and 68 (69%) received chemotherapy only as first-line systemic treatment. We were unable to record the systemic treatment protocol for 1 patient (1%). 

In total, 97% of patients had a performance status of 1 or less, and 59% were male. A total of 71% of tumors were adenocarcinoma, 75% had synchronous metastatic diseases, and in 82% of cases, the number of metastases was ≤5. There was at least one extra central nervous system (CNS) metastasis for all patients, and 71% had CNS metastasis at diagnosis. Patient characteristics are described in Table 1.

In total, 198 metastases were treated with SRT, of which 7% were lung metastases, 87% brain metastases, 4% abdominal metastases (liver or adrenal gland), 1% bone metastases, and 2% were other locations. 

For patients treated with late SRT, 61% had no progressive lesion after first-line systemic therapy, and 39% had 1 to 3 progressive lesions. For patients treated with early SRT, 68% had no new progressive lesion after first-line systemic therapy, 18% had 1 to 3 new progressive lesions, 4% had 4 or 5, and 10% had more than 5 progressive metastases.

In the early SRT group, the median time from the initiation of systemic therapy to the first SRT session was 0.8 months (interquartile range: −0.61 to 1.17 months). The first SRT session was conducted either before or within 30 days after the initiation of first-line systemic treatment for 33 patients (67%), and between 30 and 90 days for 17 patients (33%). In the late SRT group, the median time from the initiation of systemic therapy to the first SRT session was 15 months (interquartile range: 8.8 to 23.5 months).

A higher percentage of patients in the early SRT group had CNS metastases at diagnosis (94% vs. 47%). There were no other major differences between the groups.

### 3.2. Overall Survival

**Figure 1:**
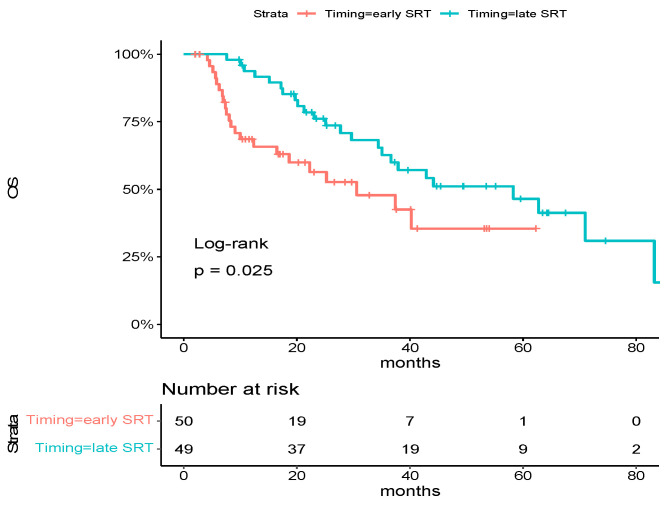
Kaplan–Meier estimates of overall survival in the overall population, according to trial group. Tick marks indicate data censored at the last time the patient was known to be alive. The hazard ratio and confidence interval were analyzed with the use of a Cox regression model.

With 46 deaths in the overall population, median overall survival was 37.9 months [95% CI: 30.6-NA]. Factors associated with a significantly poorer OS were performance status (PS) at diagnosis ≥ 2 (hazard ratio for death, 3.61; 95% CI, 1.69 to 7.70, *p* < 0.001), number of metastases at diagnosis > 3 (hazard ratio for deaths, 2.07; 95% CI, 1.13 to 3.81, *p* = 0.02) and nodal status ≥ 1 (hazard ratio for deaths, 2.36; 95% CI, 1.08 to 5.13, *p* = 0.02). There was a trend toward an improved OS for metachronous diseases (hazard ratio for deaths, 0.49; 95% CI, 0.22 to 1.05, *p* = 0.05) (Table 2). Early SRT patients had a significantly poorer OS compared to late SRT patients (median OS: 30.6 months vs. 58.3 months, hazard ratio for deaths, 1.99; 95% CI, 1.08 to 3.66, *p* = 0.02) (Figure 1). 

Factors that remained associated with OS in multivariate analysis were performance status (PS) at diagnosis ≥ 2 (hazard ratio for deaths, 2.27; 95% CI, 1.02 to 5.05, *p* = 0.04) and early SRT (hazard ratio for deaths, 1.97; 95% CI, 1.01 to 3.82, *p* = 0.046) (Table 3).

**Figure 2:**
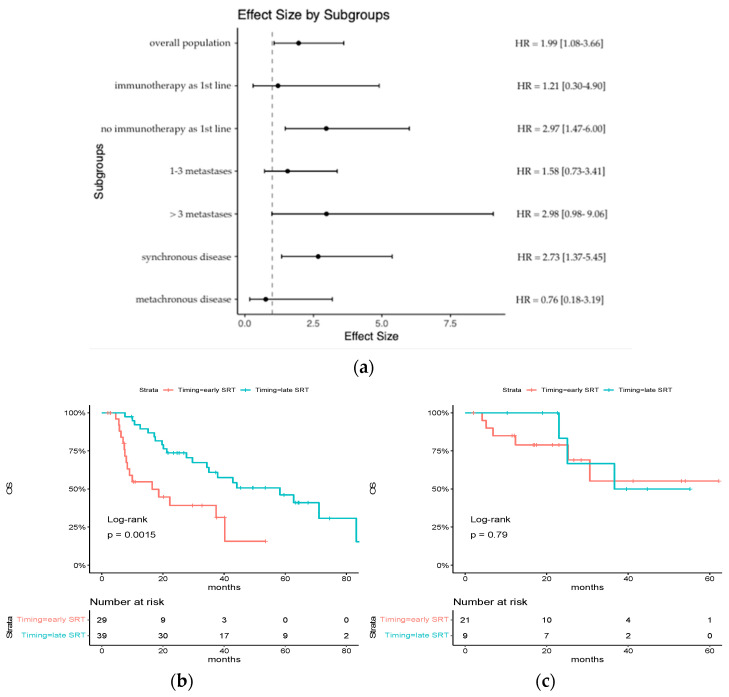
Subgroup analysis of HR for death of any cause: (**a**) an analysis of overall survival in key subgroups is shown; (**b**) Kaplan–Meier estimates of overall survival in the subgroup of patients who did not receive ICI as part of their first-line systemic treatment; (**c**) Kaplan–Meier estimates of overall survival in the subgroup of patients who received ICI as part of their first-line systemic treatment.

Subgroup analysis revealed a significant association between early SRT and poorer overall survival (OS) among patients who did not receive ICI as part of their first-line systemic treatment (median OS: 16.5 months [95% CI: 8.33-NR] vs. 58.3 months [95% CI: 35.05-NR]; hazard ratio for death: 2.97 [95% CI: 1.47–6.00]; *p* = 0.0015). For patients who received ICI as part of their first-line systemic treatment, no distinction was observed between early and late SRT (median OS: NR [95% CI: 25.2-NR] vs. 36.6 months [95% CI: 35.1-NR]; hazard ratio for death: 1.21 [95% CI: 0.30–4.90; *p* = 0.79) (Figure 2).

The hazard ratio for death also demonstrated a tendency to be higher for patients with >3 metastases at diagnosis (hazard ratio for death, 2.98 [95% CI: 0.98–9.06] vs. 1.58 [95% CI: 0.73–3.41]) and for synchronous diseases (hazard ratio for death, 2.73 [95% CI: 1.37–5.45] vs. 0.76 [95% CI: 0.18–3.19]).

### 3.3. Progression-Free Survival

Median progression-free survival in the overall population was 7.0 months [95% CI: 6.5 to 8.5]. Factors associated with a significantly poorer PFS were performance status (PS) at diagnosis ≥ 2 (hazard ratio for disease progression or death, 2.28; 95% CI, 1.09 to 4.77, *p* < 0.01) and number of metastases at diagnosis > 3 (hazard ratio for disease progression or death, 2.24; 95% CI, 1.37 to 3.67, *p* = 0.001). Early SRT patients had a similar PFS to late SRT patients (median PFS: 6.30 months vs. 7.93 months, hazard ratio for disease progression or death, 1.09; 95% CI, 0.70 to 1.68, *p* = 0.7) (Table 4). 

**Figure 3:**
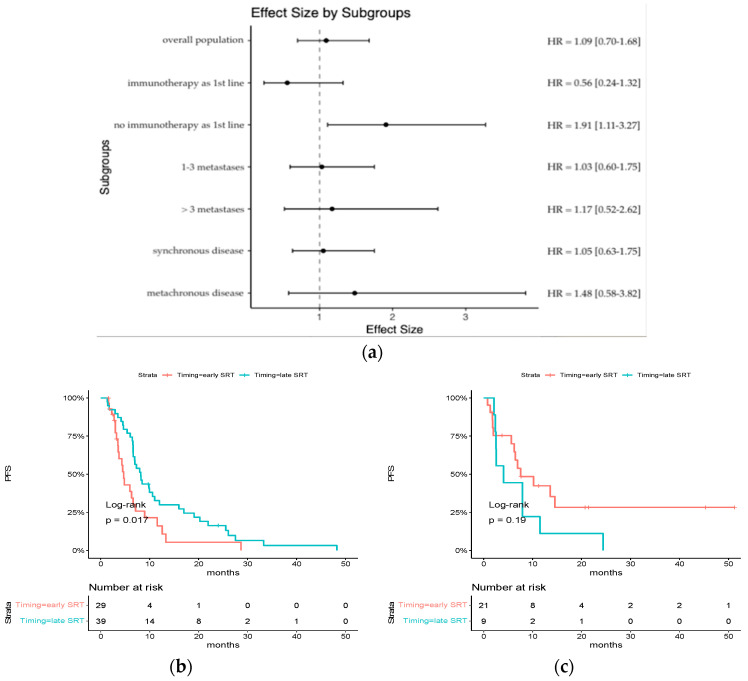
Subgroup analysis of HR for progression-free survival: (**a**) an analysis of progression-free survival in key subgroups is shown; (**b**) Kaplan–Meier estimates of progression-free survival in the subgroup of patients who did not receive ICI as part of their first-line systemic treatment; (**c**) Kaplan–Meier estimates of progression-free survival in the subgroup of patients who received ICI as part of their first-line systemic treatment.

For patients treated with chemotherapy alone, early SRT was significantly associated with a poorer PFS (median PFS: 4.69 months [95% CI: 3.57–8.98] vs. 8.20 months [95% CI: 6.66–12.00], HR = 1.91 [95% CI: 1.11–3.27], *p* = 0.017), whereas there was no difference for patients who received ICI as part of their first-line systemic treatment (median PFS: 7.54 months [95% CI: 6.23-NR] vs. 4.07 [95% CI: 2.52-NR], HR = 0.56 [0.24–1.32] *p* = 0.19) (Figure 3). The interaction effect reached statistical significance (*p* = 0.005).

There was no difference among other subgroups.

### 3.4. Time to First Subsequent Therapy

The median time to first subsequent therapy in the overall population was 10.0 months [95% CI: 7.4 to 13.7]. Factors associated with a significantly poorer TFST were performance status (PS) at diagnosis ≥ 2 (hazard ratio for subsequent therapy or death, 2.37; 95% CI, 1.14 to 4.91, *p* = 0.009), number of metastases at diagnosis > 3 (hazard ratio for subsequent therapy or death, 2.97; 95% CI, 1.76 to 4.99, *p* < 0.001), and nodal status ≥1 (hazard ratio for subsequent therapy or death, 1.79; 95% CI, 1.00 to 3.19, *p* = 0.04). Early SRT patients had a similar TFST to late SRT patients (median TFST: 9.48 months vs. 10.26 months, hazard ratio for subsequent therapy or death, 1.35; 95% CI, 0.84 to 2.17, *p* = 0.2) (Table 4). 

**Figure 4:**
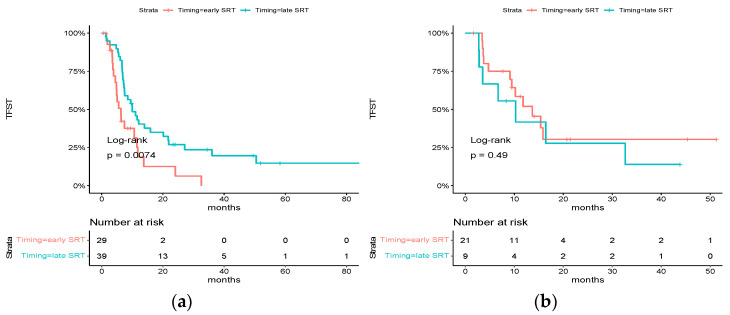
Subgroup analysis of HR for time to first subsequent therapy: (**a**) Kaplan–Meier estimates of time to first subsequent therapy in the subgroup of patients who did not receive ICI as part of their first-line systemic treatment; (**b**) Kaplan–Meier estimates of time to first subsequent therapy in the subgroup of patients who received ICI as part of their first-line systemic treatment.

For patients treated with chemotherapy alone, early SRT was significantly associated with a poorer TFST (median TFST: 6.26 months [95% CI: 4.82–11.8] vs. 10.0 months [95% CI: 7.44–21.8], HR = 2.15 [95% CI: 1.21–3.83], *p* = 0.0074), whereas there was no difference for patients who received ICI as part of their first-line systemic treatment (median TFST: 13.7 months [95% CI: 9.48–NR] vs. 10.3 months [95% CI: 3.54-NR], HR = 0.72 [0.28–1.85] *p* = 0.49) (Figure 4). An investigation of interaction was conducted, yielding a *p*-value of 0.05. 

There was no difference among other subgroups.

## 4. Discussion

For patients receiving conventional chemotherapy only as first-line systemic treatment, late SRT patients had significantly better PFS, TFST, and OS than early SRT patients. For patients receiving an ICI as part of their first-line systemic treatment, no difference in OS, PFS, and TFST was observed. Therefore, it can be hypothesized that the relevance of waiting for response evaluation to first-line systemic therapy before considering local ablative therapy could depend on the prescribed systemic treatment.

Our results support the design selected for previously published prospective trials. In the two randomized trials by Gomez et al. [7,8] and Lyengar et al. [9], radical LAT was performed only for patients who did not experience progression after FLST by platinum-based doublet. On the other hand, in the prospective trial by Bauml et al. [16], SRT was performed 4 to 12 weeks prior to the initiation of ICI treatment, while in the PEMBRO-RT/MDACC trial [15], SRT was performed concurrently or less than one week before ICI initiation. They also align with expert opinions regarding the optimal timing of SRT [13].

For patients receiving conventional chemotherapy alone, the better survival outcomes recorded for late SRT patients suggest that delaying local treatment is an effective approach to identify and exclude poorer prognosis progressive tumors. Assuming that tumors with a better prognosis are more likely to benefit from SRT [13], these findings may support the consideration of offering consolidative local treatment after evaluating systemic treatment response, rather than prior to or at the initiation of systemic therapy. 

The retrospective design, however, precludes inferring which therapeutic intervention (i.e., specific timing of SRT) can be attributed to improved survival outcomes for individual patients. Presently, only one active randomized trial exists (registered as OITROLOC, NCT 02076477) that sought to investigate the optimal timing for ablative radiotherapy in patients with synchronous oligometastatic disease. In this trial, all patients were administered systemic treatment, but they were randomly allocated to receive radiotherapy (targeting the primary tumor and metastatic sites) either at the outset or following the completion of chemotherapy. This differs from our trial, where patient selection in the late SRT group is presumed to be the primary explanatory factor for the observed differences in survival.

OS data also indicate that the significance of delaying local treatment to filter out rapidly growing tumors is particularly relevant for tumors with a poorer prognosis: high burden (>3 metastases) and synchronous diseases. In cases of high-burden diseases with a large number of lesions, postponing SRT may also allow for a reduction in the number of metastatic sites requiring treatment in case of clinical complete response of the lesions [11].

For patients receiving an ICI, multiple hypotheses might explain the absence of survival difference between the two groups. A first explanation may be that the improved tumor control achieved with ICI treatment reduces the necessity for tumor selection. Thus, in the phase 3 KEYNOTE-189 trial [30], the disease control rate was 84.6% in the pembrolizumab combination group vs. 70.4% in the placebo combination group with response rates of, respectively, 47.6% and 18.9%. In our cohort, among patients who were treated with early SRT, we also observed a higher disease control rate among patients receiving ICI as part of their first-line treatment compared to patients treated with conventional chemotherapy only: 85.7% [95% CI: 63.7 to 96.9] vs. 51.7% [95% CI: 32.5 to 70.5]. 

In the SINDAS study [11], which focuses on tumor harboring mutations in the epidermal growth factor receptor (EGFRm), LAT is also performed without upfront systemic therapy. The authors hypothesize that there could be a relatively lower necessity to administer upfront systemic therapy in the EGFRm population, considering the high disease control rate that could be expected with tyrosine kinase inhibitor treatment. This aligns with the notion that there may be a reduced imperative to initiate upfront systemic therapy as the likelihood of disease control escalates.

Another explanation might be related to the synergistic effect of immune checkpoint inhibitor and SRT. Indeed, it has recently been considered that systemic treatment by ICI could increase the effect whereby radiotherapy at one site may lead to the regression of metastatic cancer at distant sites that are not irradiated, referred to as the abscopal effect [31,32,33,34,35,36]. It has also been postulated that there could be an optimal schedule for RT administration relative to ICI in order to maximize this effect. Although the question cannot yet be answered completely, evidence suggests that SRT should be initiated slightly before, or at least not very long after, the immune checkpoint inhibitor’s first administration [17,18,19,20,21,22,23,24,25,26,27,28,29]. In the case of SRT treatment performed too long after ICI initiation, excessive tumor-associated CD8+ T cell apoptosis may occur due to increased activation and radiosensitivity induced by ICI [18,22]. 

The limitations of this study are as follows:

First of all, the study design is retrospective, which limits the level of evidence provided because of potential bias inherent to this kind of trial. Consequently, these outcomes should be considered as exploratory and hypothesis-generating in nature. We attempted to mitigate these biases by analyzing the characteristics of our subgroups (Table 1) and by performing multivariate analysis. The only significant difference between subgroups was the prevalence of CNS metastasis at diagnosis, which was more frequent for patients treated with early SRT than for those treated with late SRT. However, CNS metastasis at diagnosis did not show any association with OS, PFS, or TFST in our cohort.

Secondly, population and treatments were quite heterogeneous. Notably, SRT was not necessarily performed on all sites of disease, as seen in previously published trials on radical locally ablative treatment. It is important to emphasize, nonetheless, that the purpose of this analysis was not to evaluate the specific benefit of radical locally ablative treatment, as this has already been demonstrated in those trials. The majority of patients also had CNS metastasis at diagnosis, and the majority of brain metastases were treated, which may limit the generalizability of our findings for patients with extra-cerebral metastases only.

The subgroup of patients treated with late SRT also mixed patients with no progressive lesions under first-line systemic treatment and patients with 1 to 3 oligoprogressive lesions under first-line systemic treatment. The CURB trial [12] recently demonstrated the benefit of LAT for oligoprogressive metastatic cancer. In this setting, LAT is delayed after evaluating the response to systemic therapy, and only patients with ≤5 progressive lesions are locally treated with SRT for all progressive sites. As we aimed to compare with patients locally treated before response evaluation to first-line systemic treatment, and as local treatment with SRT has been shown to provide a benefit for oligoprogressive metastatic lung cancer, we took a pragmatic approach to combine these two populations. 

## 5. Conclusions

In conclusion, if waiting for response evaluation to first-line systemic therapy before considering local treatment with stereotactic radiotherapy is associated with improved survival for patients treated with conventional chemotherapy, there was no difference for patients receiving an immune checkpoint inhibitor.

These results may have implications for the design of future prospective trials and potentially inform clinical practice. In the context of metastatic non-small-cell lung cancer, they suggest that delaying SRT treatment to filter rapidly growing tumors may be less necessary when immune checkpoint inhibitors are administered.

## Figures and Tables

**Table 1:** Patient characteristics. Synchronous tumors refer to cases in which the first metastasis is diagnosed within 3 months of the primary cancer. Metachronous tumors refer to cases in which the first metastasis is diagnosed more than 3 months after the diagnosis of the primary cancer. All patients had at least 1 extra CNS metastasis.

		Late SRT (49)	Early SRT (50)
Age [Min–1st Qu–Median–Mean 3rd Qu–Max]		[42.8–56.2–62.7–62.1–69.1–84.6]	[35.7–54.1–64.7–63.0–72.6–84.1]
PS	0	15 (31%)	17 (34%)
	1	34 (69%)	30 (60%)
	2	0 (0%)	3 (6%)
Sex	Male	27 (55%)	31 (62%)
	Female	22 (45%)	19 (38%)
Tumor histology	Adenocarcinoma	33 (69%)	37 (74%)
	SCC	6 (13%)	6 (12%)
	Others	9 (18%)	7 (14%)
T stage	0–1	9 (26%)	9 (17%)
	2	6 (17%)	11 (23%)
	3	9 (25%)	12 (26%)
	4	11 (32%)	16 (34%)
N stage	0	9 (22%)	16 (33%)
	1	1 (3%)	4 (8%)
	2	14 (34%)	15 (30%)
	3	17 (41%)	14 (29%)
M stage	1a	13 (27%)	3 (6%)
	1b	11 (22%)	13 (27%)
	1c	25 (51%)	33 (67%)
No. of metastases at diagnosis	1–3	37 (76%)	32 (64%)
	4–5	2 (4%)	10 (20%)
	>5	10 (20%)	8 (16%)
CNS metastases at diagnosis		23 (47%)	47 (94%)
Metastasis chronology	Synchronous (<90 days)	37 (76%)	37 (74%)
	Metachronous (>90 days)	12 (24%)	13 (26%)
Symptomatic metastasis at diagnosis		26 (53%)	25 (50%)
PDL1	0	19 (58%)	21 (51%)
	1–49	10 (29%)	8 (19%)
	50–100	5 (13%)	13 (30%)
Number of metastases treated with SRT	1	27 (55%)	23 (46%)
	2	13 (27%)	12 (24%)
	3	3 (6%)	8 (16%)
	4 or more	6 (12%)	7 (14%)
Radical treatment of the primary tumor	Surgery	8 (17%)	20 (40%)
	Radiotherapy	9 (18%)	5 (10%)
	No treatment	32 (65%)	25 (50%)
Number of progressive lesions after FLST	0	30 (61%)	34 (68%)
	1–3	19 (39%)	9 (18%)
	4–5	0 (0%)	2 (4%)
	>5	0 (0%)	5 (10%)
Systemic treatment	Chemotherapy	39 (80%)	29 (58%)
	ICI +/− Chemotherapy	9 (18%)	21 (42%)
	NR	1 (2%)	0 (0%)
		**No ICI as 1st-line (68)**	**ICI as 1st-line (30)**
Age [Min–1st Qu–Median–Mean 3rd Qu–Max]		[37.9–56.1–63.0–62.4–70.4–84.6]	[35.7–54.6–64.3–63.1–69.8–84.1]
PS	0	23 (34%)	9 (30%)
	1	43 (63%)	20 (67%)
	2	2 (3%)	1 (3%)
Sex	Male	37 (54%)	20 (67%)
	Female	31 (46%)	10 (33%)
Tumor histology	Adenocarcinoma	45 (67%)	37 (80%)
	SCC	11 (16%)	24 (3%)
	Other	11 (7%)	1 (17%)
T stage	0–1	12 (22%)	5 (18%)
	2	13 (24%)	4 (14%)
	3	12 (22%)	9 (32%)
	4	17 (32%)	10 (36%)
N stage	0	18 (29%)	7 (24%)
	1	3 (5%)	2 (7%)
	2	18 (30%)	11 (38%)
	3	22 (36%)	9 (31%)
M stage	1a	12 (18%)	4 (14%)
	1b	17 (25%)	7 (23%)
	1c	38 (57%)	19 (63%)
No. of metastases at diagnosis	1–3	47 (69%)	32 (70%)
	4–5	9 (13%)	10 (10%)
	>5	12 (18%)	8 (20%)
CNS metastases at diagnosis		45 (66%)	24 (80%)
Metastasis chronology	Synchronous (<90 days)	52 (76%)	16 (70%)
	Metachronous (>90 days)	16 (24%)	9 (30%)
Symptomatic metastasis at diagnosis		37 (54%)	13 (43%)
PDL1	0	30 (65%)	10 (33%)
	1–49	10 (22%)	8 (27%)
	50–100	6 (13%)	12 (40%)
Number of metastases treated with SRT	1	35 (52%)	15 (50%)
	2	17 (25%)	7 (23%)
	3	5 (7%)	6 (20%)
	4 or more	11 (16%)	2 (7%)
Radical treatment of the primary tumor	Surgery	17 (25%)	11 (36%)
	Radiotherapy	9 (13%)	5 (17%)
	No treatment	42 (62%)	14 (47%)
Number of progressive lesions after FLST	0	45 (66%)	18 (60%)
	1–3	20 (30%)	9 (30%)
	4–5	0 (0%)	1 (3%)
	>5	3 (4%)	2 (7%)

ICI: immune check point inhibitor; PS: performance status; SCC: squamous cell carcinoma; CNS: central nervous system; SRT: stereotactic radiotherapy; FLST: first-line systemic therapy; NR: not reported. TNM stage refers to The Eighth Edition of TNM Staging of Lung Cancer (AJCC Cancer Staging Manual, Eight Edition (2017)).

**Table 2:** Factors significantly associated with overall survival in the overall population, univariate analysis.

Factor	HR (95% CI)	*p*
PS ≥2 at diagnosis	3.61 [1.69–7.70]	<0.001
>3 metastases	2.07 [1.13–3.81]	0.02
CNS metastasis at diagnosis	1.29 [0.69–2.40]	0.4
Metachronous disease	0.49 [0.22–1.05]	0.05
N ≥1 at diagnosis	2.36 [1.08–5.13]	0.02
T ≥ 3 at diagnosis	1.19 [0.94–1.49]	0.1
Early SRT	1.99 [1.08–3.66]	0.02

HR: Hazard ratio; CI: confidence interval.

**Table 3:** Factors significantly associated with overall survival in the overall population, multivariate analysis.

Factor	HR	*p*
Early SRT	1.97 [1.01–3.82]	0.046
Metachronous disease	0.46 [0.19–1.11]	0.085
N ≥1 at diagnosis	2.21 [0.96–5.07]	0.063
PS ≥2 at diagnosis	2.27 [1.02–5.05]	0.04
>3 metastases	1.33 [0.67–2.62]	0.42

**Table 4:** Factors significantly associated with progression-free survival and time to first subsequent therapy in the overall population.

	PFS		TFST	
Factor	HR	*p*	HR	*p*
PS ≥2	2.28 [1.09–4.77]	0.01	2.37 [1.14–4.91]	0.009
>3 metastases	2.24 [1.37–3.67]	0.001	2.97 [1.76–4.99]	<0.001
CNS metastasis at diagnosis	1.19 [0.75–1.87]	0.5	1.2 [0.73–1.97]	0.5
Metachronous disease	1.06 [0.65–1.73]	0.8	0.86 [0.50–1.50]	0.6
N ≥1 at diagnosis	1.55 [0.92–2.62]	0.09	1.79 [1.00–3.19]	0.04
T ≥ 3 at diagnosis	1.05 [0.89–1.24]	0.5	1.07 [0.90–1.27]	0.4
Early SRT	1.09 [0.70–1.68]	0.7	1.35 [0.84–2.17]	0.2

## Data Availability

The data presented in this study are available on request from the corresponding author.
